# Competitive Surface Colonization of Antibacterial and Bioactive Materials Doped with Strontium and/or Silver Ions

**DOI:** 10.3390/nano10010120

**Published:** 2020-01-08

**Authors:** Andrea Cochis, Jacopo Barberi, Sara Ferraris, Marta Miola, Lia Rimondini, Enrica Vernè, Seiji Yamaguchi, Silvia Spriano

**Affiliations:** 1 Department of Health Science Università del Piemonte Orientale UPO, 28100 Novara, Italy; andrea.cochis@med.uniupo.it (A.C.); lia.rimondini@med.uniupo.it (L.R.); 2 Interdisciplinary Research Center of Autoimmune Diseases, Center for Translational Research on Autoimmune and Allergic Diseases—CAAD, 28100 Novara, Italy; 3 Department of Applied Science and Technology, Politecnico di Torino, 10129 Torino, Italy; jacopo.barberi@polito.it (J.B.); sara.ferraris@polito.it (S.F.); marta.miola@polito.it (M.M.); enrica.verne@polito.it (E.V.); 4 Interdipartimental Laboratory PolitoBIOMedLab, Politecnico di Torino, 10129 Torino, Italy; 5 Department of Biomedical Sciences, College of Life and Health Sciences, Chubu University, 1200 Matsumoto, Kasugai, Aichi 487-8501, Japan; sy-esi@isc.chubu.ac.jp

**Keywords:** antibacterial activity, cytotoxicity, silver, strontium, competition for the surface, bone

## Abstract

Nowadays, there is a large amount of research aimed at improving the multifunctional behavior of the biomaterials for bone contact, including the concomitant ability to induce apatite formation (bioactivity), fast and effective osteoblasts colonization, and antibacterial activity. The aim of this study is to develop antibacterial and bioactive surfaces (Ti6Al4V alloy and a silica-based bioactive glass) by chemical doping with strontium and/or silver ions. The surfaces were characterized by Scanning Electron Microscopy equipped with Energy Dispersive X ray Spectroscopy (SEM-EDS), X-ray photoelectron spectroscopy (XPS), and Transmission Electron Microscopy (TEM). To better focus on the cells–bacteria competition for the implant surface, in addition to the standard assays for the evaluation of the bacteria adhesion (ISO22196) and for single-cell cultures or biofilm formation, an innovative set of co-cultures of cells and bacteria is here proposed to simulate a competitive surface colonization. The results suggest that all the bioactive tested materials were cytocompatible toward the bone progenitor cells representative for the self-healing process, and that the doped ones were effective in reducing the surface colonization from a pathogenic drug-resistant strain of *Staphylococcus aureus*. The co-cultures experiments demonstrated that the doped surfaces were able to protect the adhered osteoblasts from the bacteria colonization as well as prevent the infection prior to the surface colonization by the osteoblasts.

## 1. Introduction

The dental and orthopedic implants are osseointegrated through various phases involving at first the interaction with the body fluids, then acute inflammation, cell surface colonization, the production of new bone at the implant surface, and bone remodeling. Over time, the implants may suffer from infection and the formation of a bacterial biofilm at the different stages of this process [1].

The bacterial infections represent a critical event that could affect the success of a dental or orthopedic implant. Even if the prosthetic infections occur with low frequency (<2%), they can be devastating, with high hospital costs and great discomfort for the patients; on the other side, the dental implants and external orthopedic fixation devices have a much higher frequency of infection issues (<10% and 5%, respectively) [1]. The most common infections are caused by *Staphylococcus aureus* and *epidermidis*, which are Gram-positive bacteria. There are many surface characteristics that are able to influence the adhesion of the bacteria and the subsequent development of biofilm; for example, the biofilm formation occurs more frequently on the surfaces that are rougher, more hydrophobic, and/or with a higher concentration of adsorbed proteins. The biofilm formation is the result of a system of cooperation and coordination of the gene expression among the bacteria and could be explained in four steps: adhesion of single bacteria; aggregation and growth of the bacteria; maturation of biofilm; and detachment of the bacteria from the colony, which started to spread into the surrounding [2].

Normally, the infections are systemically treated with the antibiotics, even if an effective and definitive solution is not guaranteed: the bacteria can develop resistance, and efficacy is low in the case of biofilm formation on the implants. The resistance mechanism is not yet fully known, but it is clear that biofilm should have some intrinsic properties that allow high resistance to the conventional antibiotics such as (i) the 3D dense structure composed of proteins, lipids, DNA, and polysaccharides that interfere with the drug diffusion; (ii) the slow growth of biofilm, which causes the antibiotics to be taken slowly, and this gives time to the infection to spread; and (iii) the biofilm’s phenotype, which is considered as a group of bacteria that is not affected by the antibiotics [3,4,5].

Nowadays, based on these premises, the biomedical research focuses on figuring out a way to inhibit the infections and biofilm formation with alternative antibacterial agents. The use of silver as an alternative solution has been taken into consideration. Silver is widely used in the pharmaceutical industry and in many consumer products because of two fundamental advantages: the broad spectrum of its antimicrobial activity, including both Gram-positive and Gram-negative species, and the lack of evidence associated with bacterial silver resistance. Several silver forms and compounds are currently used as antimicrobial agents: silver ions dissolved in a solution (such as silver sulfadiazine or nitrate) and silver nanoparticles. The modes of action of the silver ions (Ag^+^) are strictly linked to the ion concentration of the solution, which is based on the increase in the reactive oxygen species (ROS) production connected to an increase in the concentration of H_2_O_2_: an oxidative imbalance inside the bacteria occurs with the inhibition of proliferation. To reach this state, silver, in whatever form it is, must be able to enter through the bacterial membrane [6], and it occurs because Ag^+^ is able to create bonds with the metabolic enzymes and proteins with S-pending groups and with the nitrogen groups characteristic of the DNA chain [7]. Concerning the cytotoxicity of the silver ions and nanoparticles, conflicting results are reported in the literature. Ion doping, compared to surface functionalization through nanoparticles, allows a progressive release of silver that can be much more easily tailored, predicted, and controlled, in order to obtain an effective antibacterial activity, reducing the cytotoxicity risks. On the other hand, the use of nanoparticles is very promising, but this is less known and controllable, and it has been not used as a strategy of the present research in order to avoid potential cytotoxicity concerns [8]. A low dose of silver might have a beneficial effect on the bone formation, as it could promote the proliferation and osteogenesis of the mesenchymal stem cells and have an inhibition effect on the production of the Tumor Necrosis Factor cytokine (TNF-α), resulting in an inhibition of osteoclastogenesis and inflammation [9,10]; however, a wrong concentration can also lead to an opposite effect, resulting in strong cytotoxicity and imbalance toward the osteoclasts formation, thus interfering with the bone remodeling process [11].

Lastly, strontium is a metal with chemical similarity to calcium that is used in the form of salts as an activator of the osteoblasts due to its ability to activate the calcium receptors: it can positively contribute to reducing the risk of bacterial colonization by promoting an early adhesion of the osteoblasts and balancing an eventual cytotoxic reaction to silver [12].

Considering all that is written above, the rationale of this study is to develop antibacterial and bioactive surfaces (Ti6Al4V alloy and a silica-based bioactive glass) by chemical doping with strontium and/or silver ions and to test the competitive surface colonization of the osteoblast progenitors and bacteria biofilm. The specimens were investigated for their physical–chemical properties by means of SEM-EDS, XPS, and TEM analyses.

Firstly, the biological characterization was performed by means of the standard assays for the antibacterial and cytocompatibility evaluation involving the use of osteoblasts and bacteria in separate environments; afterwards, to simulate the surface competition, the osteoblasts and bacteria were cultivated together in the same environment using three different approaches to test whether the performed surface doping was able to promote cells’ adhesion and spread over bacterial contamination.

## 2. Materials and Methods

### 2.1. Sample Preparation

Ti6Al4V alloy plates (TITANIUM Consulting & Trading S.r.l., Firenze, Italy) were treated starting from the procedure described in [13]. At first, they were polished with 400 grid abrasive papers. The treatment consists of soaking in 5 M NaOH solution and then in a mixed solution of 50 mM CaCl_2_ and 50 mM SrCl_2_; reagents from Kanto Chemical Co., Inc., Tokyo, Japan_._ They were heat treated at 600 °C for 1 h, followed by soaking in a 1 M Sr(NO_3_)_2_ solution doped with 1 mM AgNO_3_; reagents from Kanto Chemical Co., Inc., Tokyo, Japan_._. The resultant products were denoted by Ti64(Sr+Ag). Polished Ti6Al4V alloy samples were used as control for the biological tests (undoped Ti6A4V alloy).

A bioactive glass in the system SiO_2_–Na_2_O–CaO–P_2_O_5_–B_2_O_3_–Al_2_O_3_ (SBA2- all reagents from Sigma-Aldrich, St. Louis, MO, USA) was prepared and used as control (un-doped glass) in the biological tests; this glass was already prepared and characterized by the authors, and some data are reported in [14,15]. The bioglass samples were prepared by melt and quenching technique in bar forms, annealed, cut into slices, and polished with 1000 grid abrasive papers. The doped samples (denoted by SBA2-Ag) were soaked in a 30 mM AgNO_3_ solution to incorporate the silver ions on their surface.

All the Ti6Al4V alloy, Ti64(Sr+Ag) alloy, and SBA2 or SBA2-Ag specimens were prepared as 1 cm side and 1 mm thick squares; prior to the biological assays, they were heat sterilized (3 h, 100 °C) and stored at room temperature until use.

### 2.2. Transmission Electron Microscopy Equipped with Energy-Dispersive Spectroscopy (TEM-EDS)

TEM lamellae were prepared from Ti64(Sr+Ag) and SBA2-Ag samples using an FEI Helios NanoLab Focused (ThermoFisher Scientific, Waltham, MA, USA) ion beam/scanning electron microscope. The platinum coating that can be seen in the Energy Dispersive X-ray Spectroscopy (EDS) maps is deposited as a protective buffer during Focused Ion Beam (FIB) preparation.

The TEM data were acquired on an FEI Tecnai Osiris (ThermoFisher Scientific- Waltham, MA, US) operated at 200 kV. Imaging and diffraction were carried out on an Ultrascan Charged Coupled Device (CCD) camera. The EDS data were acquired in Scanning Transmission Electron Microscopy (STEM) mode using a large area EDS detector (0.9 sr) and processed using a proprietary software and Hyperspy—an open source Python-based suite for the electron microscopy analysis. The interplanar distances were calculated by using Powder Diffraction Standards (JCPDS) as reference.

### 2.3. SEM-EDS

The surfaces of Ti64(Sr+Ag) and SBA2-Ag samples that had been subjected to carbon coating were observed under field emission scanning electron microscopy (FE-SEM: S-4300, Hitachi Co., Tokyo, Japan) with a voltage of 15 kV. Their chemical composition was examined with an energy-dispersive X-ray spectrometer (EDX: EMAX-7000, Horiba Ltd., Kyoto, Japan) by using 9 kV-K that penetrate approximately 1 μm in depth. Five areas were analyzed, and the averaged value was used.

### 2.4. XPS

The chemical composition of the surfaces of Ti64(Sr+Ag) and SBA2-Ag samples was also analyzed by X-ray photoelectron spectroscopy (XPS; XPS, PHI 5000 Versaprobe II, ULVAC-PHI, Inc., Kanagawa, Japan) using an Al–Ka radiation line as the X-ray source. The take-off angle was set at 45° so that the system is able to detect photoelectrons to a depth of 1–5 nm from the surface. The binding energy of the obtained spectra was calibrated by reference to 284.6 eV of the C1s peak, which is attributed to the CH_2_ groups. The peak profile analysis of the Ag region was performed by using the National Institute of Standards and Technology (NIST) database: Ag^+^-20667-12-3; Ag^0^ 7440-22-4. The peak profile analysis of the Ag region obtained on a metal Ag foil is reported as a reference.

### 2.5. Ion Release in Fetal Bovine Serum (FBS)

Ion release in fetal bovine serum (FBS) was performed through soaking the samples in 2 mL of FBS (without any dilution). At different time points, respectively 1 and 6 h, 1, 3, 7, 14, 21, and 28 d, the samples were removed from the serum and placed into a fresh new medium. The FBS at each time point was stored and frozen until the analysis. The cumulative ion release for each sample was calculated at each point (from 6 h on) by means of addition of the ion release value at the selected time point to the previous ones. The ion release cumulative curves were obtained by averaging the cumulative curves of each sample.

The FBS samples were mineralized by acid digestion in a microwave oven (Milestone MLS-1200 Mega microwave laboratory unit, Gemini, Apeldoorn, The Netherlands). Sample aliquots of 500 µL were treated with 800 µL of nitric acid in tetrafluoromethoxyl (TFM) bombs (Sigma-Aldrich, St. Louis, MO, USA). Six heating steps (1 min at 250 W, 2 min at 0 W, 5 min at 200 W, 5 min at 350 W, 5 min at 550 W, and 5 min at 250 W), followed by a ventilation step of 10 min, were applied; the second step at 0 W for the digestion procedure is necessary to avoid the eventual exothermic reaction between the sample and acid reagent (nitric acid). At the end of the treatment, the resulting solutions were diluted to 2.3 mL with high-purity water (HPW).

Ag concentration was determined by a graphite furnace absorption spectrometer (GF-AAS), model 5100 (Perkin Elmer, Waltham, MA, USA) equipped with a Zeeman-effect background correction, an HGA 600 graphite furnace, and an AS-60 autosampler. Pyrocoated graphite tubes with an L’Vov platform and hollow cathode lamp for Ag were used throughout. The external calibration method and a matrix modifier (0.015 mg Pd and 0.01 mg Mg(NO_3_)_2_—Sigma-Aldrich, St. Louis, MO, USA) were employed. The volume sampled into the graphite tube was 20 µL for the test sample and calibration solutions. The volume of the matrix modifier was 10 µL.

Sr determination was performed by an inductively coupled plasma optical emission spectrometer (ICP-OES), model Optima 7000 DV (Perkin Elmer, Waltham, MA, USA), equipped with a Mira Mist nebulizer, a Cyclonic spray chamber, and a double monochromator (prism and Echelle grating). The instrumental conditions were: plasma power 1.3 kW, sample aspiration rate 1.5 mL min^−1^, argon nebulizer flow 0.6 L min^−1^, argon auxiliary flow 0.2 L min^−1^, and argon plasma flow 15 L min^−1^. The wavelength used for Sr was 407.771 nm, and an external method calibration was employed for its determination.

HPW produced with a Millipore Milli-Q system was used throughout. All the reagents used were of analytical grade. Standard Ag and Sr solutions were prepared from concentrated stock solutions (Sigma-Aldrich, St. Louis, MO, USA).

The selection of two different analytical techniques for the two elements is based on their sensitivity. In fact, ICP sensitivity for Ag is limited and not suitable for the Ag concentrations investigated here. This is the reason why GF-AAS was used for silver. On the other hand, the sensitivity of ICP for Sr is higher, thus, it is suitable for Sr determination in the conditions investigated here. This is the reason why it was used in this work for Sr determination. It must be underlined that the results of the two techniques are fully comparable.

### 2.6. Direct Cytocompatibility Evaluation

#### 2.6.1. Cells Culture Conditions

The in vitro cytocompatibility of the Ti6Al4V alloy and bioactive glass specimens was tested toward human progenitor osteoblasts (hFOB 1.19, ATCC CRL-11372, from the American Type Culture Collection, Manassas, Virginia, USA) that were selected as representative for bone self-healing. The osteoblasts were cultivated following the manufacturer’s instructions using Dulbecco’s modified eagle medium and F12 medium mix (50:50, both from Sigma-Aldrich, St. Louis, MO, USA), supplemented with 10% fetal bovine serum (FBS), 1% antibiotics (penicillin/streptomycin), and 0.3 mg/mL neomycin (G418 from Sigma-Aldrich, St. Louis, MO, USA).

#### 2.6.2. Cells Metabolic Activity Evaluation

One centimeter and 1 mm thickness square sterile specimens (both bioactive glasses and Ti alloys) were gently located by sterile tweezers on the bottom of the wells of a 12 multiwell plate (2.2 cm diameter), avoiding any surface damage. Then, osteoblasts were dropwise (100 μL) seeded directly onto the specimens’ surface at a defined number (1 × 10^4^/sample), allowed to adhere for 2 h at 37 °C; afterwards, they were rinsed with 1 mL of fresh medium and cultivated for 1 to 3 days. At each time point, specimens were moved to a new 12-well plate, and the osteoblasts metabolism was evaluated by the colorimetric Alamar blue assay (alamarBlue™ from Life Technologies, Milan, Italy) using a spectrophotometer (Spark, Tecan, Basel). Briefly, the ready-to-use Alamar solution was added to each well containing the test specimens (1 mL/specimen), and the plate was incubated in the dark for 4 h at 37 °C in the incubator to allow resazurin dye reduction into fluorescent resorufin upon entering living cells. Then, an aliquot of 200 μL of the supernatant was collected and centrifuged to remove any debris (1200 rpm, 2 min); 100 μL were finally spotted into a new black-bottom 96-well plate to minimize auto-signaling. A fluorescent signal was evaluated at 570 nm wavelength, and the results presented as relative fluorescent units (RFU); alamar solution (intended as cells-free) fluorescence was considered as blank and subtracted to the test samples results.

### 2.7. Direct Antibacterial Evaluation

#### 2.7.1. Strain Growth Conditions

The specimens’ antibacterial activity was evaluated toward a pathogenic drug-resistant (Methicillin-Oxacillin) certified strain of *Staphylococcus aureus* (*S. aureus* ATCC 43300, purchased from the American Type Culture Collection, Manassas, VA, USA). The bacteria were cultivated following the manufacturer’s instructions: briefly, the strain was seeded overnight into a selective mannitol salt agar plate (Sigma-Aldrich, St. Louis, MO, USA) until round single colonies were formed. Then, 2–3 colonies were collected and resuspended into 10 mL of a Luria Bertani broth (LB, from Sigma-Aldrich, St. Louis, MO, USA) and incubated in agitation (120 rpm) at 37 °C for 18 h to reach the logarithmic growth phase. Finally, the broth culture was diluted using a fresh LB medium until the optical density was 0.001 at 600 nm, corresponding to 1 × 10^5^ cells/mL. Fresh LB medium was used as a blank for Optical Density (O.D.) evaluation; the obtained bacteria suspension was assayed with the colony-forming units (CFU) count to verify the density of the inoculum.

#### 2.7.2. Antibacterial Evaluation

Two different protocols were used to assay the specimens’ antibacterial properties: the first one corresponds to the ISO 22196 standard, and it is optimized to evaluate the materials’ surface properties in contact with a bacterial suspension [16], while the second one has been previously published by the authors, and it is designed to force the biofilm formation and test surface efficacy toward the latter [17,18,19].

For ISO 22196 protocol, 1 cm and 1 mm thickness square sterile specimens (both bioactive glasses and Ti alloys) were gently located by sterile tweezers on the bottom of the wells of a 12-multiwell plate, and then, 100 μL of the bacterial suspension was directly dropped onto the specimens’ surface and covered with a sterile polyethylene film. The inoculated specimens were placed in an incubator at 35 °C for 24 h. After incubation, each specimen was washed with a soybean casein digest broth containing lecithin and polyoxyethylene sorbitan monooleate (SCDLP broth, 5 mL) to recover the bacteria. Then, the colony-forming units (CFU) were counted as follows: an aliquot of 100 μL of the supernatant was collected from each specimen and used to perform six-serial 10-fold dilutions, mixing 20 μL of the bacterial suspension with 180 μL of the sterile saline (0.9% NaCl). Then, 20 μL were collected from each dilution, spotted onto the plates containing the LB agar medium, and incubated for 24 h at 37 °C. The CFU ml^−1^ were counted as follows [20]:

CFU = [(number of colonies × 10)^(serial dilution)]

where

number of colonies = countable single round colonies;
10 = the ratio between the total volume (1 mL) and the 100 µL used for the 10-fold dilutions;
serial dilution = 1–6 10-fold dilution areas where colonies were counted.

In the second assay (named by now as UPO), the 1 cm and 1 mm thickness square sterile specimens (both bioactive glasses and Ti alloys) were gently located into a 12-multiwell plate by sterile tweezers, avoiding any surface damage. Then, each specimen was fully submerged with 1 mL of LB broth containing 1 × 10^5^ bacteria prepared as prior described in Section 2.7.1; the plate was incubated at 37 °C into an orbital shaker for 90 min in agitation (120 rpm) to allow the separation between the adherent biofilm bacteria and non-adherent floating planktonic bacteria (separation phase). After 90 min of agitation, the plate was recovered, and the supernatants containing the planktonic bacteria were removed and replaced with 1 mL of fresh media to cultivate only the surface-adhered biofilm selected during the separation phase (growth phase). So, the surface-adhered biofilms were grown at 37 °C for 24 h prior to evaluation. The number of viable biofilm colonies was counted by the CFU method as prior described.

Finally, the results were expressed by means of the R score, which was calculated according to the ISO 22196 instructions as follows:

(R) score = log_10_ (T_24(cnt)_/T_24(test)_)

where

T_24(cnt)_ = number of colonies in controls after 24 h
T_24(test)_ = number of colonies in test after 24 h.

### 2.8. Co-Cultures of Osteoblasts and Bacteria

**Figure 1:**
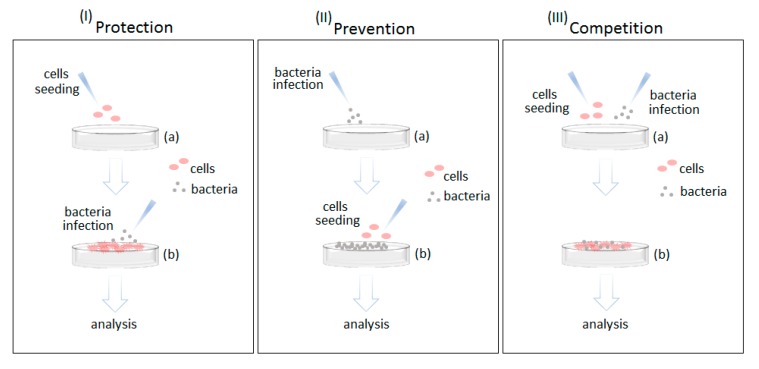
Schematic representation of the three cells–bacteria co-culture methods.

To reproduce a osteoblasts–bacteria competition for the surface colonization, three different protocols were designed to simulate as many conditions as schematized in Figure 1: (i) protection, (ii) prevention, and (iii) competition. For each assay, 1 cm and 1 mm thickness square sterile specimens (both bioactive glasses and Ti alloys) were gently located into a 12-multiwell plate by sterile tweezers, avoiding any surface damage.

#### 2.8.1. Protection Conditions

In the protection condition, it was simulated an infection in the implant site toward a surface undergoing cells’ colonization in the healing process (schematized in Figure 1, left panel). Accordingly, the cells (1 × 10^4^/specimen) were seeded directly onto the sterile specimens and allowed to adhere and spread for 24 h in a complete medium (intended as Dulbecco’s modified eagle medium (DMEM)/F12, 10% FBS, 1% antibiotics, 0.3 mg/mL neomycin). The day after, the medium was removed and replaced with 1 mL of fresh antibiotic-free DMEM/F12 10% FBS medium previously infected with 1 × 10^5^ bacteria prepared as described in Section 2.7.1 (using the medium as blank for O.D. evaluation). After 24 h of direct contact between osteoblasts and bacteria, the specimens were washed three times with PBS; then, trypsin was applied (5 min, 37 °C) to detach the osteoblasts and adhered biofilm bacteria. After detachment, the supernatant was collected, and viable osteoblasts number was counted by trypan blue using a Bürker chamber, while bacteria viable colonies were detected by CFU count as prior described.

#### 2.8.2. Prevention Conditions

In the prevention condition, an infection occurring in the implant site was simulated prior to the cells starting to colonize the surface to undergo healing (schematized in Figure 1, central panel). Accordingly, the bacteria were seeded directly onto the specimens’ surface at 1 × 10^5^ cells/mL concentration in order to develop a biofilm layer as described in the UPO protocol. After 24 h of biofilm growth, the osteoblasts were seeded directly onto the pre-contaminated surfaces in a defined number (1 × 10^4^/sample) using the antibiotic-free medium. After 24 h of direct contact, the specimens were washed three times with PBS, and the bacteria and osteoblasts number were evaluated as prior described in the protection condition.

#### 2.8.3. Competition Conditions

The competition condition was simulated to examine how the cells and bacteria compete for the same surface when they are seeded together (schematized in Figure 1, right panel). Accordingly, the osteoblasts (1 × 10^4^) and bacteria (1 × 10^5^) were mixed together in 1 mL of an antibiotic-free medium and seeded directly onto the specimens’ surface. In this setup, the concentration of cells and bacteria was kept the same as that of the prevention and protection conditions previously discussed, but it must be considered that the results of competition will be strongly affected by the inoculum concentration, as deeply discussed in the results and discussion section. After 24 h of direct contact, the specimens were washed three times with PBS, and the bacteria and osteoblasts number were evaluated by the same methods used in the previous conditions.

### 2.9. Statistical Analysis

All experiments were performed in triplicate.

Concerning ion release, the standard deviations of the cumulative ion release values were calculated by pooling the ones relative to each sample [21].

The data on the cytocompatibilty and antibacterial tests were analyzed by using SPSS software (v25, IBM, New York City, NY, USA) by means of one-way ANOVA followed by Tukey’ test as post hoc analysis. The significance level was set at *p* < 0.05.

## 3. Results

### 3.1. Surface Chemical Composition, Crystalline Structure, and Morphology

**Figure 2:**
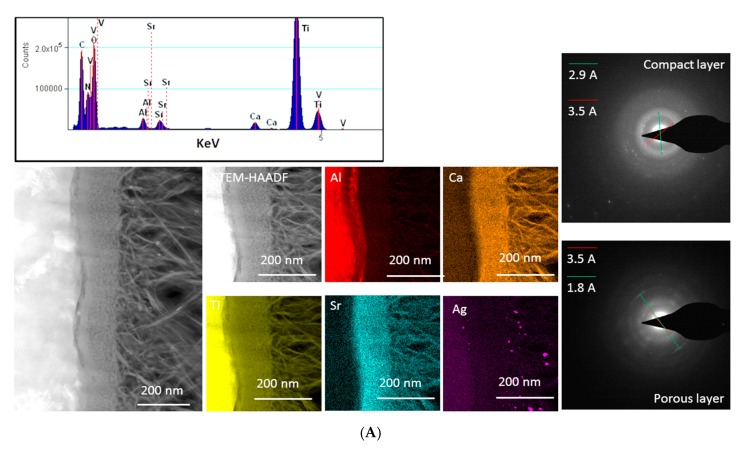

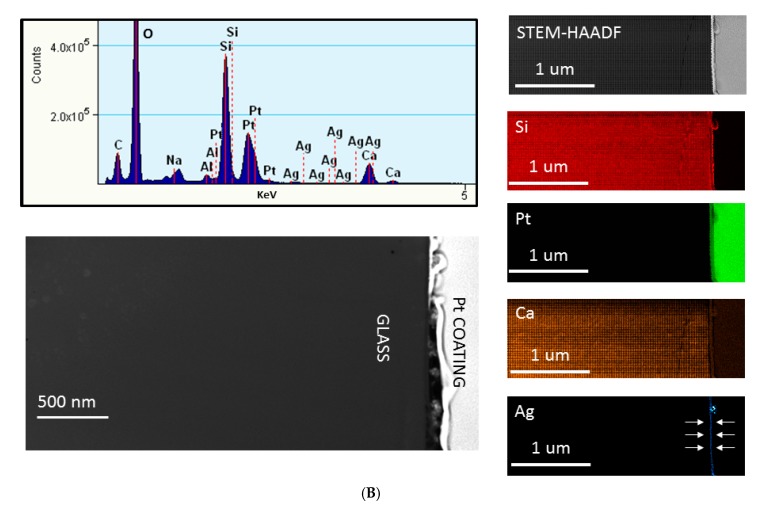
TEM-EDS data and images obtained on the cross-sections of Ti64(Sr+Ag) (**A**) and SBA2-Ag (**B**).

**Figure 3:**
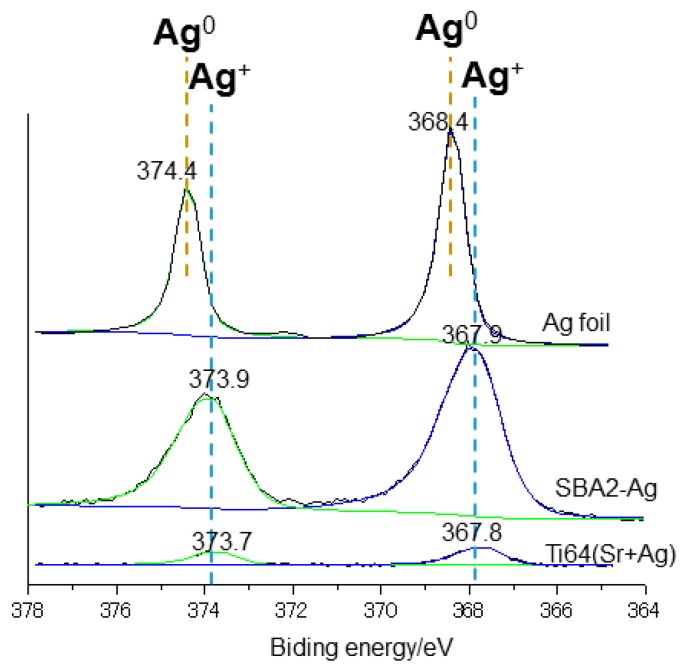
XPS peak profile analysis of the Ag region obtained on Ti64(Sr+Ag) and SBA2-Ag. The peak profile analysis of the Ag region obtained on a metal Ag foil is reported as reference.

Some data coming from a chemical and physical characterization of Ti64(Sr+Ag) and SBA2-Ag were previously reported by the authors [13,14,15]. In the present research, morphological, structural, and chemical characterization was performed through TEM-EDS (on the cross-sections), SEM-EDS, and XPS analyses (on the top surfaces), in view of a better understanding of the biological results obtained in the same research; the results are reported and compared in Figure 2 and Figure 3 and Table 1.

Concerning the surface crystalline structure and morphology, the two materials are different. Ti64(Sr+Ag) shows a crystalline surface oxide layer, about 1.5 μm in thickness, with a compact inner and a fibrous outermost layer with a porous appearance on the nanoscale (Figure 2A, TEM cross-section). The diffraction pattern obtained on the compact layer (d_hkl_ = 2.9–3.5 Å) might be attributed to anatase, brookite, and CaTi_2_O_5_; while the one obtained on the porous layer (d_hkl_ = 3.5–1.8 Å) might be attributed to anatase and CaTi_2_O_5_. SBA2-Ag has an amorphous structure and shows a smooth surface (Figure 2B, TEM cross-section).

Concerning the surface composition, it was analyzed by EDS and XPS (Table 1). EDS analysis refers to about 1–2 µm penetration depth, while it is of a few nanometers in the case of XPS, and a smaller volume of material, confined on the outermost layer, is analyzed in this case. According to the different penetration depth of the EDS and XPS analyses, as well as to the expected high relative error for the elements at low concentration, the obtained value can be compared on a semi-quantitative basis. In the case of Ti64(Sr+Ag), Ag, Ca, O, and Sr are detected in a larger amount by XPS, according to their higher concentration in the surface oxide layer, while it is the opposite for Al and Ti, which are in higher concentration in the substrate. In the case of SBA2-Ag, the greater difference between the two analyses is registered in the case of Ag and Ca: it can be expected that these elements have a different distribution across the thickness of the material. Comparing the presence of the antibacterial agents in the two materials, comparable results concerning the amount of silver are obtained by SEM-EDS analysis on the top surfaces of both the materials (Table 1): 0.2 at% on Ti64(Sr+Ag) and 0.3 at% on SBA2-Ag. Moreover, Ti64(Sr+Ag) also contains the Sr^2+^ ions (about 1.5 at% by SEM-EDS on the top surface). SBA2-Ag shows a consistent higher amount of silver than Ti64(Sr+Ag) when analyzed through XPS, and the difference between XPS and EDS data can be related to the distribution of silver: silver is concentrated in the outermost surface layer (a few nanometers) of the glass, while it is much more distributed within a layer of micrometric thickness in the case of the metal.

The XPS peak profile analysis of the silver region (Figure 3) evidences a doublet at 367.9 and 373.9 eV for SBA2-Ag and a doublet at 367.8 eV and 373.7 eV, which can be both associated with silver in the ionic form according to the literature data [22] and the NIST database. As a reference, an Ag metal foil was analyzed, and it is reported in Figure 3: a clear shift of the silver peak in the case of SBA2-Ag and Ti64(Sr+Ag) with respect to metallic silver can be observed, while the peaks of metallic silver are not detected.

The distribution of silver and strontium within the surface layer was investigated through TEM-EDS chemical maps on the cross-sections. As expected, silver is limited to a very thin surface layer in the bioactive glass SBA2-Ag. The signal of silver in the surface layer of Ti64(Sr+Ag) in the EDS map is weaker than on the previous material, while it is evident that strontium is homogeneously distributed across all of the oxide layer. Ag particles were not observed on the glass sample (SBA2-Ag) by TEM observations, and only a few were observed on Ti64(Sr+Ag) (Figure 2A,B), suggesting that Ag was mainly incorporated as ions in agreement with the XPS analyses previously reported.

Both the surfaces of the alloy and glass samples are bioactive and able to form hydroxyapatite during soaking in simulated body fluid, similar to that formed on Ti metal subjected to the same solution and heat treatment, as already investigated and reported [14,15,23].

### 3.2. Ion Release in FBS

Soaking in a fluid rich in protein (FBS) was performed up to 28 days for the Ag-doped materials and samples of the solutions collected and analyzed at fixed time steps in order to evaluate the kinetics of the silver and strontium ion release in a condition close to the physiological one. FBS was selected in order to take into account the great affinity of the proteins (mainly albumin) with the silver ions. The silver(I) ions have high affinity for the ligands, which are polarizable and, considering all the functional groups of a protein, binding is expected with the sulfur ligand on the side-chain of cysteine and that of methionine [24].

**Figure 4:**
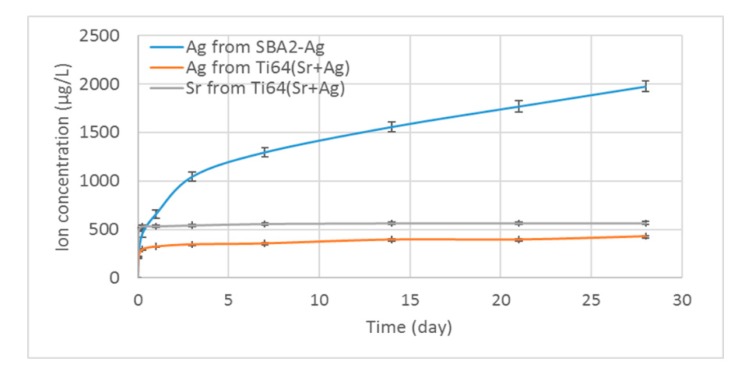
Ion release of Ti64(Sr+Ag) and SBA2-Ag in fetal bovine serum (FBS) during 28 days of soaking.

The results of silver quantification in FBS are reported in Figure 4.

As first, both materials have a fast release of ions in the first day of soaking. Ti64(Sr+Ag) releases most of the silver and strontium ions during the first day of soaking with a maximum value of about 300 and 550 µg/L for Ag^+^ and Sr^2+^ ions, respectively. SBA2-Ag releases about 650 and 1050 µg/L of Ag^+^ in the first and third day of soaking, respectively. Ion release slowly goes further over time for Ti64(Sr+Ag), while SBA2-Ag is able to continuously release the silver ions along all the tested period (28 days) with a maximum value of about 2000 µg/L at the end of soaking.

### 3.3. Cytocompatibility Evaluation

**Figure 5:**
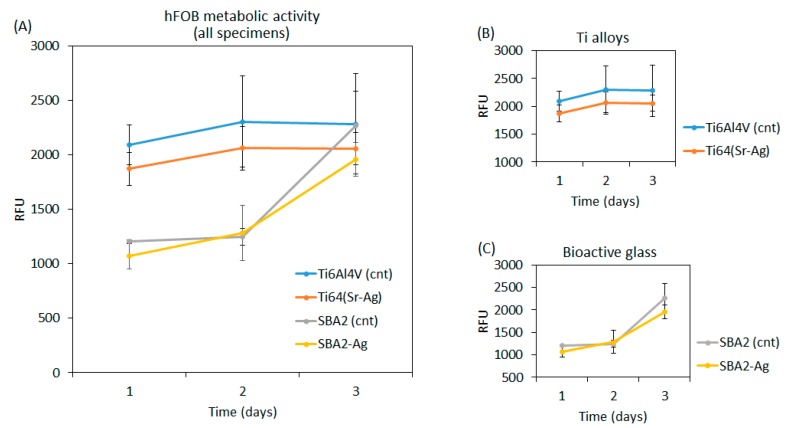
Evaluation of cytocompatibility of both the raw (cnt) and doped (Ag) specimens. In general (**A**), no statistically significant differences were noticed in the cells metabolism (*p* > 0.05) in detail, nor for the Ti alloy (**B**) nor bioactive glass (**C**) the Ag-doping determined toxicity.

In order to evaluate the biological response of the tested materials, first, a cytocompatibility assay was performed in vitro using human osteoblasts progenitors (hFOB). The results are reported in Figure 5.

The introduction of the Ag^+^ ions did not reduce the cytocompatibility of both the Ti alloy and bioactive glass, as no significant difference was noticed between the raw control materials (cnt) and doped ones (Figure 5A, *p* > 0.05). Looking at the details of the Ti alloy (Figure 5B) and bioactive glass (Figure 5C), a different trend was noticed in the metabolic activity of the osteoblasts. The titanium alloys (doped and control) show a little increase of the cell number up to 48 h, reaching a plateau after 72 h. The bioactive glass (both with or without doping with silver) displayed a reduced number of osteoblasts in the first 2 days, which was probably due to the high surface reactivity typical of a bioactive material with a fast kinetic of ion release, followed by a fast increase up to 72 h. The results were comparable at day 3, thus suggesting a similar osteoblasts number and metabolic activity after 72 h in direct contact with the tested materials.

### 3.4. Antibacterial Evaluation

**Figure 6:**
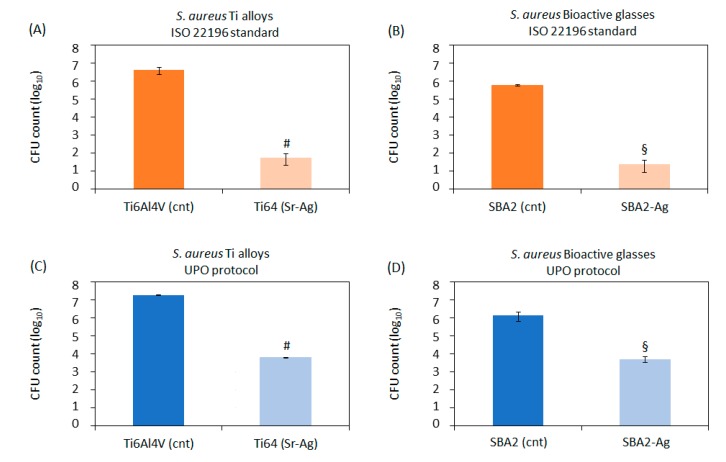
Antibacterial evaluation according to ISO standard (upper panel—(**A**,**B**)) and UPO protocol (lower panel—(**C**,**D**)). Both the assays demonstrated that the Ag-doped specimens were able to significantly reduce the bacteria number in comparison with the untreated controls (*p* < 0.05, indicated by # and §, respectively). The bars represent means ± standard deviations.

As first, a standard test made by following ISO22196 standard protocol, which was performed toward *S. aureus* MultiDrug-Resistant (MDR) in order to evaluate the antibacterial activity of the doped surfaces with respect to the control ones (SBA2 and polished Ti6Al4V alloy). The results are reported in Figure 6A,B. A statistically significant difference (*p* < 0.05, indicated by # and §, respectively) was noticed by comparing the controls (cnt) and doped materials (Ti64(Sr+Ag) and SBA2-Ag), while the two doped surfaces have similar behavior. A quantitative evaluation of the antibacterial activity of the tested materials is described later in the text by using the R score.

In order to investigate the specific behavior of the doped materials with respect to biofilm formation, *S. aureus* MDR biofilm was adhered and cultivated directly onto the specimens’ surface by the UPO protocol. The results are reported in Figure 6C,D. In this test, the bacteria are seeded in a defined number on the test specimens (infection stage); then, the samples are incubated in agitation for a fixed time (separation stage). During this step, the mechanical forces due to the media floating cause a physical separation between the biofilm and the planktonic counterparts (biofilm formation stage). In fact, the bacteria genetically programmed to adhere to a surface successfully attach to the materials’ surface, while, on the opposite, bacteria not programmed for adhesion (planktonic) float into the liquid supernatant. So, by removing the supernatant after the separation phase, it is possible to cultivate only the biofilm attached to the surface for the desired time point(s) (biofilm growth stage). In this case as well, a statistically significant difference (*p* < 0.05, indicated by # and §, respectively) was noticed by comparing the controls (cnt) and doped materials (Ti64(Sr+Ag) and SBA2-Ag), evidencing that these materials can effectively fight biofilm formation on their surfaces.

The R score was calculated, as suggested by ISO22196 (that is the difference between the average of the common logarithm of the number of the viable bacteria recovered from the untreated control specimens and doped ones after 24 h); the results obtained on the different materials tested by the two protocols (ISO22196 and biofilm formation) are reported in Table 2 as well as the count of the initial inoculum (O.D. = 0.001, named as T0) that was confirmed in the magnitude of 1E5.

### 3.5. Co-Culture of Osteoblasts and Bacteria

Further biological tests (co-cultures) were applied in order to evaluate how the doped surfaces can act with respect to the competition between the osteoblasts and bacteria for the surface colonization.

Accordingly, here, three methods were designed to simulate (i) an infection of a healing implant (*protection*), (ii) an insertion into a contaminated site (*prevention*), and (iii) a competitive challenge for the surface of a newly implanted device (*competition*).

**Figure 7:**
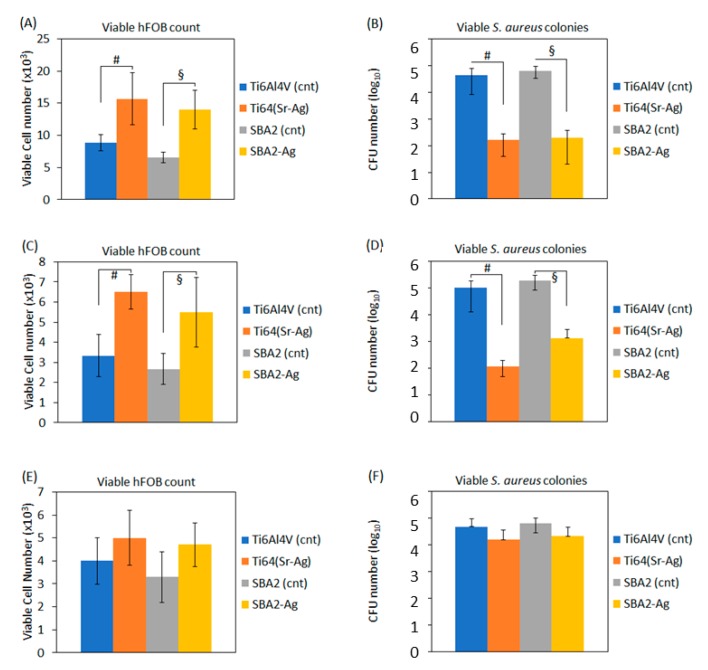
The results from the osteoblasts-bacteria co-cultures for the protection (**A**,**B**), prevention (**C**,**D**) and competition (**E**,**F**) methods. The surface treatments were able to both protect osteoblasts ((**A**) *p* < 0.05 vs. cnt, indicated by # and §) and prevent them from infection ((**C**), *p* < 0.05 vs. cnt, indicated by # and §) by significantly lowering the number of S. aureus CFU ((**B**,**D**), *p* < 0.05 vs. cnt, indicated by # and §). However, in the competition method, Ag doping did not report significant differences in comparison with controls for both viable osteoblasts and bacteria CFU count ((**E**,**F**), *p* > 0.05). Bars represent means and standard deviations.

The first method is here named as “protection”, and the obtained results are reported in Figure 7A,B. The osteoblasts previously seeded onto the materials’ surface (1 × 10^4^) were found to be increased even after 24 h of infection on the Ag-doped surfaces, showing a significant difference in comparison with the raw control materials (Figure 7A, *p* < 0.05, indicated by # and §, respectively) where the cell number was lowered from the seeding. As confirmation, the number of the viable *S. aureus* colonies was significantly reduced by the surface Ag doping in comparison with the untreated materials (Figure 7B, *p* < 0.05, indicated by # and §, respectively), thus showing clearly an opposite effect of the surfaces toward the osteoblasts or bacteria.

The second method here named “prevention” was designed by an environment rich in bacteria, and the results are reported in Figure 7D (*p* < 0.05, indicated by # and §, respectively). After 24 h of growth onto the infected specimens, the number of the viable cells was significantly higher onto the Ti64(Sr+Ag) and SBA2-Ag-doped specimens in comparison with the untreated controls (Figure 7C, *p* < 0.05, indicated by # and §, respectively).

Finally, a third protocol here named “competition” was designed following the hypothesis of the “race” (competition) for the surface, as postulated in 1987 by Gristina [25]. In order to respect the same experimental conditions within all the assays, we applied a 1 × 10^5^ bacteria/specimen infection in the competition protocol also. After 24 h of competition, some differences were noticed between the control and test specimens for both the viable osteoblasts count (Figure 7E) and *S. aureus* CFU number (Figure 7F), but the results were not statistically significant (*p* > 0.05).

## 4. Discussion

According to the rationale of this study to develop antibacterial and bioactive surfaces by chemical doping with strontium and/or silver ions, the chemical state, amount, and distribution of these elements on the surfaces were investigated first.

According to the preparation techniques of these materials, it is expected that Ag is introduced into the surfaces mainly through ion exchange with Ca^2+^ ions in Sr-containing calcium titanate for Ti64(Sr+Ag) and with Na^+^ ions in amorphous SiO_2_ for SBA2-Ag. Thus, it is expected that Ag^+^ ions were surrounded by TiO_2_ or SiO_2_ after introduction into the surfaces. The ionic state of silver is confirmed by XPS profiles of Ag 3d, which are different from metallic Ag and close to the Ag^+^ ions reported in the literature [26] as well as in the NIST database. A few silver nanoparticles were observed by TEM on Ti64(Sr+Ag), but they seem to involve a minority fraction of silver. This is in agreement with the strategy of this research which—unlike the other surface treatments of Ti alloys explored by the authors [27]—is focused on the antibacterial action of ions, avoiding the risks of uncontrolled release due to nanoparticles.

According to all the analyses performed, it can be concluded that both silver and strontium are homogeneously dispersed within the surface oxide layer of Ti64(Sr+Ag). Silver is not detected in the TEM-EDS chemical map because of its lower amount with respect to strontium and because it is widely distributed within the entire oxide layer. Differently, silver is highly concentrated in the outermost surface layer in the case of SBA2-Ag. This is why it is detectable in the TEM-EDS chemical map, even if its total amount in EDS analysis is not far from that of Ti64(Sr+Ag) (as confirmed by XPS analyses).

The ion release data are of great interest. Both silver and strontium are released from the surfaces when in contact with fluids. The release of silver is of interest with respect to antibacterial behavior. Strontium was added mainly because of its therapeutic effect on the osteoporotic bone, which it achieves by increasing new bone formation while reducing bone resorption [28]: its release from the surface of Ti64(Sr+Ag) during the time is promising and meets the expectations. Both materials have a fast release of ions in the first day of soaking, which is expected and useful in case of the early infections occurring in the first hours after surgery. A long-term silver release extended up to about one month is not usual in silver-doped materials [27], and it is of interest in order to avoid late infections. Second, it is interesting that each material–ion pair has its own release capability, and the amount of the ions released is not simplistically correlated to the surface amount of the elements on the different samples. For instance, it was not expected that Ti64(Sr+Ag) has a similar release of Sr^2+^ and Ag^+^ ions considering their different amount on the outermost surface of the sample or that SBA2 has a significantly higher release of Ag^+^ ions than Ti64(Sr+Ag) considering their similar amount in the EDS analysis across a thicker surface section. The different crystalline structure and surface morphology of the two materials can play a role in this respect, and they will be better investigated in the future. Concerning crystallographic structure and surface morphology, the two materials differ because Ti64(Sr+Ag) is crystalline and highly porous, while SBA2-Ag is amorphous and smooth. In the case of Ti64(Sr+Ag), the initial fast release may be attributed to release from the grain boundaries of the calcium titanate crystals containing Sr^2+^ and Ag^+^, while the latter’s slow release may be attributed to the diffusion of ions from inside the crystals [13]. The role of the proteins in the mechanism of silver and strontium release from these materials will have to be further investigated in the future, as well as the long-term release of silver at high concentration from an amorphous matrix where silver is concentrated on the outermost layer.

The biological characterization was firstly performed by means of the standard assays for the antibacterial and cytocompatibility evaluation. Cells deputed for the self-healing process (hFOB) were selected as representative for the specific clinical application, and *Staphylococcus aureus* was selected as frequently related with the bone infections. The introduction of the Ag^+^ ions does not reduce cytocompatibility toward the osteoblasts of both the Ti alloy and bioactive glass with respect to the analogous undoped materials. Referring to the ions release evaluation results, the here-obtained range was previously described as toxic for in vivo studies, as reported for the example by van der Zande et al., who showed that even a lower Ag^+^ ion accumulation in blood was toxic for rats [29]. In fact, ions were recovered in almost all organs after 28 days of administration. Actually, the release values obtained here are probably overestimated when referred to over a long time period because they were collected in a small volume of fluid and cannot be directly compared to the in vivo tests: further in vivo analysis is strongly necessary in the future to verify the biocompatibility of the materials tested here.

The applied doping resulted in antibacterial surfaces with great effectiveness both with respect to the bacterial direct adhesion on the surface (ISO22196) and biofilm formation (UPO protocol): the tested materials can be considered as antibacterial according to the international standard and can effectively fight biofilm formation on their surfaces.

However, it should be noted that the ions release results (Figure 4) reported a great difference between bioactive glasses and Ti alloys that the antibacterial results did not report. This can be justified by the fact that antibacterial assay is limited to 24 h when the difference in ion release is not so marked: so, it can be supposed that longer time points could have highlighted a higher efficacy from the bioactive glasses that are able to release a significantly higher amount of ions as a function of time in comparison with the treated Ti alloy. In addition, a contribution from the Sr^2+^ ions to the antibacterial action can be considered in the case of Ti64(Sr+Ag), as reported in [30,31].

The obtained results are in agreement with the minimum inhibitory concentration (MIC) value reported in the literature, which is 30 µg/L [32]. The R score shows that the protocol of ISO 22196 is a more sensitive assay than that of the biofilm formation for surface property evaluation. The differences obtained by comparing ISO and UPO protocols can be ascribed to the fact that the UPO method is based on a forced separation between biofilm and planktonic populations (the separation phase detailed in Section 2.7.2), while in the ISO one, a mixed biofilm + planktonic bacterial population is tested. It is well known that the biofilm counterpart is much more resistant to the antibacterial treatments than the floating planktonic one, so it was probably more resistant to the Ag^+^ ions released from the Ti and bioglass specimens’ surface. Accordingly, this is probably the reason why lower results were obtained by applying the same materials in the UPO protocol in comparison to the ISO standard. However, the comparison between doped and raw materials was significant also in the more severe test, thus suggesting that the tested materials are of great relevance in order to avoid the drawbacks coming from the implant surface contamination from antibiotic-resistant biofilm such as the explant and revision surgeries.

Afterwards, the osteoblasts and bacteria were cultivated together in the same environment using three different approaches to test whether the performed surface doping was able to promote cell adhesion and spread over bacterial contamination. The first method here named as “protection” probably resembles the most frequent clinical scenario where patients that underwent an orthopedic surgery and bone implantation are subjected to a postoperative infection during the late healing. Nowadays, the secondary infections are more and more frequent, thus representing a huge problem for patients due to the need of a second surgery to recover the device fixation. To give an example, Zhang et al. [33] recently reviewed fractures of the tibia and fibula diagnosed in Zhengzhou No. 7 People’s Hospital from 2007 to 2016, showing that 52/100 patients required a second surgery due to late infection. The authors’ conclusion was obviously that improvement on the implantable devices is strongly required to prevent these late infections. Here, encouraging results were obtained on the tested doped materials. The osteoblasts seeded onto the materials’ surface were found to be increased even after 24 h of infection on the Ag-doped surfaces, showing a significant difference in comparison with the raw control (undoped) materials where the cell number was lowered from the seeding. As confirmation, the number of the viable *S. aureus* colonies was significantly reduced by the surface Ag doping in comparison with the untreated materials, thus showing clearly an opposite effect of the surfaces toward the osteoblasts or bacteria.

The second method here named “prevention” was designed by an environment rich in bacteria to resemble the clinical scenario where the bone device is implanted into patients that present an infected anchorage site. For example, in orthopedics, this is the case of the peri-prosthetic joint infection (PJI), which is a condition affecting about 1% of patients carrying hip implants [34]. This condition requires complex and protracted treatments, sometimes leading to the need for an excision arthroplasty or amputation. The gold-standard treatment of PJI consists of the two-stage revision proposed by Fitzgerald and Jones in 1985, where the artificial hip joint is removed, and the replacement is delayed for several months until a clear evidence of infection eradication is obtained [35]. However, the fast increase of drug-resistant strains led to the re-infection of about 8% of the hip implants following the two-stage surgical revision, as recently reviewed by Kunutsor et al. from the analysis of 98 clinical studies [36]. So, it is strongly required that the implanted devices hold intrinsic antibacterial properties to counteract those bacteria that resist the antibiotic therapy. The Ag-doped materials that previously demonstrated strong antibacterial properties in the single culture assay confirmed the ability to be able to significantly reduce the number of viable *S. aureus* colonies that were firstly seeded onto the specimens’ surface in comparison to the untreated control materials, thus allowing adhesion and proliferation of the osteoblasts that were subsequently seeded onto the pre-infected surfaces. These results are very promising to speculate that these surface treatments were able to permit the osteoblasts adhesion and proliferation within an infected environment.

Finally, a third protocol here named “competition” was designed following the hypothesis of the “race” (competition) for the surface, as postulated in 1987 by Gristina [25], where it is supposed that the fate of a biomaterial surface depends on the competition between the tissue and bacteria for the implant surface. Following this hypothesis, some works are reported in the literature involving the bone-dedicated materials and combining, for example, the human osteosarcoma (U2OS or K12) cells and *Staphylococcus epidermidis* or *Staphylococcus aureus* [37], the mouse embryonic pre-fibroblasts (MC3T3-E1), and *Staphylococcus epidermidis* [38], but the results are not easy to interpret. In fact, most of the results coming from the present literature suggest that the outcome of the study depends mainly by the bacterial inoculum concentration used at the start of the competition [37,39,40,41]; in fact, considering a concentration range from 1 × 10^2^ to 1 × 10^5^ bacteria/specimen, only the lower inoculum led to significant results between antibacterial and control surfaces. These results logically correlate the improvement of the aseptic surgery conditions and pharmacological prophylaxis that can dramatically lower the ratio of the infection with the success of implantology due to the intrinsic ability of the biomaterials to counteract the low rate of infections. Our results are in line with those findings: in fact, the results were not statistically significant. The differences in terms of loss in antibacterial activity of the doped specimens can be probably also ascribed to a higher aggregation and precipitation due to the high protein content of the medium and the presence at the same time in the same environment of cells and bacteria. In fact, it was demonstrated that Ag^+^ ions aggregation is maximized in relation to the medium composition, and thus their metabolism interference toward bacteria or cells is significantly lowered in that scenario [42].

In conclusion, we can affirm that both the surfaces have a similar behavior concerning cytocompatibility (with respect to osteoblast progenitor cells) and antibacterial action (with respect to *S. Aureus)*. The different structure, composition, morphology, and ion release rate of the two doped surfaces do not induce a different biological response in the tested conditions. Moreover, an innovative experimental procedure is here reported that is able to test the antibacterial ability of the surfaces of interest for the bone implants, as well as their cytocompatibility and to evaluate the competitive surface colonization. The results suggest that all the bioactive tested materials are biocompatible (hFOB 1.19) and the doped ones are antibacterial (*Staphylococcus aureus*), as well as able to inhibit biofilm formation both if the bacteria come in contact with the surface before or after the osteoblast progenitors.

## 5. Conclusions

The surface doping of Ti6Al4V alloy and a bioactive glass with strontium and/or silver ions resulted in in vitro cytocompatible materials in direct contact with human osteoblast progenitor cells responsible for the bone self-healing. The introduction of silver has conferred strong antibacterial properties to the surfaces, making them effective at preserving osteoblasts and counteracting an infection in the co-cultures assays where the osteoblasts and bacteria were cultivated in the same environment.

## Figures and Tables

**Table 1:** X-ray photoelectron spectroscopy (XPS) and SEM-EDS analysis obtained on the top surfaces of Ti64(Sr+Ag) and SBA2-Ag.

Sample	Analysis	Element/at%									
		Na	O	Ti	Ag	Sr	Ca	P	Al	V	Si
Ti64(Sr-Ag)	XPS	-	67.4	25.4	0.8	2.7	2.9	-	0.8	0.1 *	-
SBA2-Ag	XPS	5.7	67.3	-	4.2	-	0.3	0.0 **	1.4	-	21.0
Ti64(Sr-Ag)	EDS	-	64.9	29.6	0.2	1.5	1.9	-	1.3	0.7	-
SBA2-Ag	EDS	11.0	60.5	-	0.3	-	9.5	2.7	0.4	-	15.4

* Ti is overlapped with V; ** P is overlapped with B.

**Table 2:** R score values calculated by the colony-forming unit (CFU) count at the seeding day (T(O)) and after 24 h of culture in direct contact with the specimens’ surface (T(24)).

Sample	Protocol	T(0) CFU Count	T(24) CFU Count	(R) Score
Ti6Al4V (cnt)	ISO 22196	4.88 × 10^5^	3.98 × 10^6^	
Ti64 (Sr+Ag)	ISO 22196	4.88 × 10^5^	5.68 × 10	4.8
Ti6Al4V (cnt)	UPO	4.88 × 10^5^	1.78 × 10^7^	
Ti64 (Sr+Ag)	UPO	4.88 × 10^5^	6.08 × 10^3^	3.4
SBA2	ISO 22196	4.88 × 10^5^	5.58 × 10^5^	
SBA2-Ag	ISO 22196	4.88 × 10^5^	2.38 × 10	4.3
SBA2	UPO	4.88 × 10^5^	1.38 × 10^6^	
SBA2-Ag	UPO	4.88 × 10^5^	5.18 × 10^3^	2.4
